# Effect of Live Classical Music in Homeless and Hospital Settings

**DOI:** 10.7759/cureus.117394

**Published:** 2026-10-03

**Authors:** Joseph S Wang, Nader Wehbi, Natalie Briggs, Helena Yip, Esteban Hernandez Parra

**Affiliations:** 1 Biology, Catalina Foothills High School, Tucson, USA; 2 Department of Otolaryngology - Head and Neck Surgery, University of Arizona, Tucson, USA; 3 College of Liberal Arts and Sciences, Arizona State University, Tempe, USA; 4 School of Music, University of Arizona, Tucson, USA

**Keywords:** classical music, homeless persons, hospital environment, housing insecurity, listening to music, mood and anxiety, music intervention, short stai, stai questionnaire

## Abstract

Introduction

The therapeutic effects of classical music in reducing stress have been well documented, particularly in healthcare settings. However, classical music has also been used to discourage unhoused individuals from remaining in public spaces. For example, loud classical music is sometimes played at gas stations and train stations to deter unhoused individuals from lingering in these areas. Our study sought to answer the question of whether exposure to live classical music in an unhoused or homeless population would lead to a similar reduction in anxiety levels compared with staff, patients, and patients’ families in a hospital setting.

Methods

Brief anonymous surveys were based on the State-Trait Anxiety Inventory (STAI), a validated anxiety questionnaire. A shortened five-item survey was administered to participants seated in the cafeteria of the hospital and also in dining areas of facilities serving the unhoused. A single musician played the violin/viola for 60 minutes, while audience members were asked to rate themselves on a scale of 1 to 4 in categories including content, worried, tense, calm, and upset before and after listening to at least 15 minutes of classical music.

Results

Two groups were surveyed, including the Hospital Group (N = 47) as the comparison group and the Unhoused Group (N = 66). A Student’s t-test was used for analysis. Classical music was associated with improved feelings in both groups. When comparing change scores between groups, improvements were similar for “calm” (p = 0.092), “tense” (p = 0.678), “upset” (p = 0.183), and “content” (p = 0.123). “Worried” feelings decreased significantly more in the Unhoused Group than in the Hospital Group (p = 0.037). When stratified by gender or duration of housing insecurity, there was no difference in the improvement scores.

Conclusions

This observational study suggests that exposure to live classical music is associated with a self-reported short-term improvement in anxiety levels in the unhoused population.

## Introduction

The therapeutic effect of classical music has been well studied and documented in stress-related situations [1], such as the healthcare setting [2,3]. Stress is a response to physical or emotional challenges. The response can also be physical, emotional, or behavioral. Being sick as a patient or caring for someone who is sick, whether as a family member or a healthcare worker, can lead to stress. The prevalence of hospitalized patients has been shown to be 28% in a meta-analysis [4]. The so-called “Mozart effect,” or playing recorded classical music, has been shown to reduce anxiety, pain medication requirements, and heart rates and blood pressure in patients [5-7]. Anxiety is also common in healthcare workers. The prevalence of anxiety has been shown to be 23.3% among healthcare workers, vs. 19.1% among non-healthcare workers, using data from the 2021-2024 National Health Interview Survey [8].

Despite being largely studied as a stress-mitigating tool, classical music has been used as a deterrent to the unhoused population in public spaces [9]. Classical music is played at loud volumes to discourage unwelcome stays [10]. Classical music broadly refers to Western art music composed from approximately the 11th century to the present day. It is characterized by its use of Western instruments, formal compositional structures, and notation systems that allow for precise reproduction across different performances. It ranges from chamber music to the standard symphony orchestra, which includes four main sections: strings (violins, violas, cellos, and double basses), woodwinds (flutes, oboes, clarinets, and bassoons), brass (trumpets, horns, trombones, and tubas), and percussion (timpani, cymbals, and triangles) [11].

A survey of the literature in PubMed yielded limited studies on music therapy for veterans with housing insecurity or music group activities to foster community among veterans or homeless youth [12,13], but they did not address whether exposure to classical music could have a beneficial effect on anxiety levels for these individuals. According to the American Psychiatric Association, “anxiety is an emotion characterized by apprehension and somatic symptoms of tension in which an individual anticipates impending danger, catastrophe, or misfortune. The body often mobilizes itself to meet the perceived threat: muscles become tense, breathing is faster, and the heart beats more rapidly” [14]. Being unhoused may be one of the most stressful situations a human can experience, as having shelter is a basic human need [15]. The prevalence of anxiety in homeless persons can range from 36% to 51%, severe enough to interfere with functioning [16]. The terms “homeless” and “unhoused” can be used interchangeably, but for consistency’s sake, this manuscript will use the term “unhoused.” Housing insecurity is also another term used in this manuscript to denote unstable housing, which could include intermittently being homeless or staying in a hotel, car, shelter, or with relatives or friends. This study was performed to determine whether the same beneficial effects on anxiety from classical music exposure in hospital settings could be extended to the unhoused population.

## Materials and methods

Study population

The study population included two groups: one comprising people in the hospital setting, which could include staff, family members, and patients, and a second group comprising people experiencing housing insecurity. The study started on January 18, 2026, and ended on February 21, 2026.

Eligibility criteria

The inclusion criteria were anyone over the age of 18 who was willing to participate. The exclusion criteria were anyone who was not proficient in English, had hearing deficits, or was unwilling to participate.

Study design and setting

Live classical music was performed on the violin and viola while surveys were administered at the women’s and men’s shelters of the Tucson Primavera Foundation and the Z Mansion, which provided free food, clothing, and basic medical care to the unhoused population of Tucson. The Z Mansion hosts a brunch for unhoused individuals every Sunday morning and provides basic medical screening, clothing, and hygiene supplies. The brunch serves approximately 200 people, based on the number of available tables and chairs. The women’s and men’s shelters accommodate approximately 20 to 30 individuals each at a time. The addresses of the Primavera Foundation shelters were withheld for privacy reasons.

The repertoire included unaccompanied violin and viola pieces, Bach Cello Suites Nos. 2 and 3, the first and second movements of the Bruch Violin Concerto No. 1, and the first movement of the Stamitz Viola Concerto. The musician did not interact with the participants. There was one concert at each location, including the hospital cafeteria, the Z Mansion, the men’s shelter, and the women’s shelter. The participants were not the same at each location. The paper surveys were handed out by two peers of the musician, who gave a brief explanation of the survey.

Sampling technique

A random sampling technique was used. The surveys were given out while participants were eating a meal. For the comparison population, participants seated in the cafeteria at Banner University Medical Center Tucson were surveyed while listening to live classical music performed by the primary author.

Instruments and data collection

Brief anonymous surveys were based on the State-Trait Anxiety Inventory (STAI), a validated anxiety questionnaire [17]. A shortened five-item survey was used, similar to the six-item survey used in the study by Tluczek et al., except for omitting the “I feel relaxed” item. In the Tluczek study, a cohort of 288 parents of newborns with normal and abnormal newborn screens was evaluated using a short form, which was highly correlated with the 20-item STAI score, and all internal consistency reliabilities were greater than 0.90 [18]. Over the years, multiple short forms have been developed to minimize participant fatigue, and they have been validated as well. The scores were not tallied into a total per survey. Scores from each category from 1 to 4 were compared pre- and post-exposure within groups and across the two groups. The survey also asked for limited demographic information, including age, gender, familiarity with classical music, and favorite genres of music. For surveys of the unhoused population, the duration of housing insecurity was queried. For surveys conducted in the hospital setting, participants were asked whether they were patients, family members, or staff. The survey had five different categories: calm, tense, upset, content, and worried. Participants were asked to rate their levels in each category on a scale of 1 to 4 before and after hearing the primary author play his violin and viola for at least 15 minutes. The actual exposure duration for each participant was 15+ minutes, but the actual exposure time was not standardized. The minimum duration was set based on the observed time of eating for diners in the hospital cafeteria. The time for participants in the unhoused facilities was at least an hour, as they were also receiving distributions of hygiene supplies and clothing. The exact time of exposure to music was not enforced or tracked. Participants were asked to time themselves. Participants were allowed to leave in the middle of the performance.

Ethical consideration

Institutional Review Board (IRB) approval for the study was obtained through the IRB committee of the Southern Arizona Science and Engineering Fair. All participants surveyed were informed verbally that their participation was entirely voluntary. There was a written paragraph about voluntary participation on the survey, but signed consent was waived because the signature itself could compromise the anonymous nature of the survey, and the IRB committee allowed the waiver of signatures. There was no financial compensation for completion of the survey. This research did not receive any specific grant from funding agencies in the public, commercial, or not-for-profit sectors.

Data analysis

Statistical analysis was performed within the same group using a paired Student’s t-test in IBM SPSS Statistics for Windows, version 30.0 (released 2024; IBM Corp., Armonk, NY, USA) to compare the different categories of anxiety before and after exposure to music in the Hospital and Unhoused Groups. The independent-samples t-test was used to compare changes in values between the Hospital and Unhoused Groups and for stratification according to gender and familiarity with classical music. ANOVA was used to analyze subgroups within the Unhoused Group based on duration of housing insecurity. P < 0.05 was considered statistically significant.

## Results

The Hospital Group included 47 participants, 21 female and 26 male, aged 21-74 years (median = 40). In terms of familiarity with classical music, 36 answered yes, and 11 answered no. Fifteen people were family members, 29 were staff members, and three declined to state. There were no patients captured in this single-session study. The genres of preferred music included pop, rock, Latin, country, and gospel, but none included classical music (Table 1).

**Table 1:** Demographics of Hospital and Unhoused Groups.

Group	N	Female	Male	Age range	Familiarity with classical music
Hospital Group	47	21	26	21-74	36 yes
Unhoused Group	66	25	41	21-75	50 yes

The Unhoused Group included 66 participants, 25 females and 41 males, aged 21-75 years (median = 49.5). In terms of familiarity with classical music, 50 answered yes, 11 answered no, and five declined to state. Six people reported experiencing housing insecurity for less than a year; 15 people reported between one and two years; 12 people reported between three and five years; 15 people reported more than five years; and 18 people declined to state. The genres of preferred music included pop, rock, Latin, country, gospel, Jewish, and Spanish, but none included classical music.

Surveys were considered complete if the short five-item STAI survey was completed. Demographic information was partially collected on most, but not all, surveys. Therefore, stratification analyses based on gender or housing status only included participants who had completed the five-item STAI short form as well as the queried demographic items.

The sample size for the participants at the unhoused facilities over the three performances at the various sites was 270. The sample size for the participants in the hospital cafeteria over a one-hour period was 120.

The number of people approached in the Unhoused Group was 194, and the number of people approached in the hospital was 72. Four surveys were discarded from the Hospital Group due to an incomplete five-item short form, and six surveys were discarded from the Unhoused Group due to an incomplete five-item short form. The number of eligible participants completing pre- and post-surveys was 47 for the Hospital Group and 66 for the Unhoused Group. The reasons for nonparticipation and noncompletion were unknown. The response rate calculated was 70.8% in the Hospital Group and 37% in the Unhoused Group.

Six people reported experiencing housing insecurity for less than a year; 15 people reported between one and two years; 12 people reported between three and five years; 15 people reported more than five years; and 18 people declined to state.

Classical music was associated with improved feelings in both the Unhoused and Hospital Groups (Tables 2, 3).

**Table 2:** Effect of classical music on feelings - Unhoused Group (N = 66). Paired Student’s t-test was used for the analysis.

Feelings	Before classical music	After classical music	Mean change (after-before)	P-value
Calm	2.92	3.3	0.38	0.021
Tense	2.34	1.61	-0.73	<0.001
Upset	2.03	1.45	-0.58	<0.001
Content	2.68	2.95	0.27	0.073
Worried	2.44	1.58	-0.86	<0.001

**Table 3:** Effect of classical music on feelings - Hospital Group (N = 47). Paired Student’s t-test was used for the analysis.

Feelings	Before classical music	After classical music	Mean change (after-before)	P-value
Calm	2.6	3.36	0.77	<0.001
Tense	2.11	1.53	-0.57	0.001
Upset	1.49	1.21	-0.28	0.017
Content	2.62	3.17	0.55	0.002
Worried	1.98	1.57	-0.4	0.021

In the Unhoused Group, “calm” increased significantly (P = 0.021), and “tense,” “upset,” and “worried” feelings decreased significantly (P < 0.001). “Content” feelings increased, but this was not statistically significant (P = 0.073) (Table 2).

When comparing change scores between groups, improvements were similar for “calm” (P = 0.092), “tense” (P = 0.678), “upset” (P = 0.183), and “content” (P = 0.123). “Worried” feelings decreased significantly more in the Unhoused Group than in the Hospital Group (P = 0.037) (Table 4).

**Table 4:** Comparing change scores between Unhoused (N = 66) and Hospital Group (N = 47). Independent-samples t-test was used for the analysis.

Feelings	Unhoused Group mean change	Hospital Group mean change	Mean difference	P-value
Calm	0.38	0.77	-0.39	0.092
Tense	-0.73	-0.57	-0.16	0.678
Upset	-0.58	-0.28	-0.3	0.183
Content	0.27	0.55	-0.28	0.123
Worried	-0.86	-0.4	-0.46	0.037

At baseline (before music), the Unhoused Group had higher reported levels of “upset” (P = 0.003) and “worried” (P = 0.046) than the Hospital Group, but there were no significant baseline differences in “calm,” “tense,” or “content” feelings (all P-values > 0.05) (Table 5).

**Table 5:** Baseline scores of feelings comparing Unhoused vs. Hospital Groups. Independent-samples t-test was used for the analysis.

Feelings	Unhoused Group mean before	Hospital Group mean before	Mean difference	P-value
Calm	2.92	2.6	0.32	0.088
Tense	2.34	2.11	0.23	0.239
Upset	2.03	1.49	0.54	0.003
Content	2.68	2.62	0.06	0.666
Worried	2.44	1.98	0.46	0.048

Stratified analyses showed no significant differences in either group based on gender (all P-values > 0.05) (Tables 6, 7).

**Table 6:** Feelings in Unhoused Group stratified by gender pre- and post-music exposure. Independent-samples t-test was used for the analysis.

Feelings	Male mean pre (n = 38)	Male mean after (n = 38)	Male mean change	Female mean pre (n = 25)	Female mean post (n = 25)	Female mean change	P-value
Calm	3.05	3.39	0.34	2.72	3.16	0.44	0.831
Tense	2.29	1.58	-0.71	2.48	1.68	-0.8	0.381
Upset	2.11	1.32	-0.79	1.96	1.68	-0.28	0.121
Content	2.81	3.08	0.27	2.44	2.72	0.28	0.609
Worried	2.45	1.45	-1	2.48	1.8	-0.68	0.449

**Table 7:** Feelings in Hospital Group stratified by gender pre- and post-music exposure. Independent-samples t-test was used for the analysis.

Feelings	Male mean pre (n = 25)	Male mean after (n = 25)	Male mean change	Female mean pre (n = 22)	Female mean post (n = 22)	Female mean change	P-value
Calm	2.68	3.4	0.72	2.5	3.32	0.82	0.958
Tense	2.12	1.6	-0.52	2.09	1.45	-0.64	0.722
Upset	1.48	1.2	-0.28	1.5	1.23	-0.27	0.663
Content	2.6	3.24	0.64	2.64	3.09	0.45	0.485
Worried	1.96	1.64	-0.32	2	1.5	-0.5	0.503

Stratification by familiarity with classical music showed no differences for most outcomes, with one exception: In the Unhoused Group, changes in “upset” feelings differed significantly depending on how familiar the respondent was with the classical music genre (P = 0.024).

Unhoused participants who said they were not familiar with classical music reported a greater decrease in feelings of “upset” after listening to classical music (Tables 8, 9).

**Table 8:** Feelings of Unhoused Group stratified by familiarity with classical music pre- and post-exposure. Independent-samples t-test was used for the analysis.

Feelings	Familiarity yes pre (n = 50)	Familiarity yes after (n = 50)	Mean change	Familiarity no pre (n = 11)	Familiarity no after (n = 11)	Mean change	P-value
Calm	2.94	3.38	0.44	2.91	2.91	0	0.459
Tense	2.32	1.72	-0.6	2.45	1.27	-1.18	0.127
Upset	1.92	1.5	-0.42	2.64	1.36	-1.27	0.024
Content	2.68	2.92	0.24	2.6	2.9	0.3	0.703
Worried	2.4	1.56	-0.84	2.73	1.82	-0.91	0.937

**Table 9:** Feelings of Hospital Group stratified by familiarity with classical music pre- and post-music exposure. Independent-samples t-test was used for the analysis.

Feelings	Male mean pre (n = 36)	Male mean after (n = 36)	Male mean change	Female mean pre (n = 11)	Female mean after (n = 11)	Female mean change	P-value
Calm	2.68	3.4	0.72	2.5	3.32	0.82	0.958
Tense	2.12	1.6	-0.52	2.09	1.45	-0.64	0.722
Upset	1.48	1.2	-0.28	1.5	1.23	-0.27	0.663
Content	2.6	3.24	0.64	2.64	3.09	0.45	0.485
Worried	1.96	1.64	-0.32	2	1.5	-0.5	0.503

Stratification according to duration of housing insecurity also did not show any differences between subgroups (Table 10).

**Table 10:** Feelings of Unhoused Group based on duration of housing insecurity. ANOVA was used for the analysis.

Feelings	<1 year (n = 6)	1-2 years (n = 15)	3-5 years (n = 12)	>5 years (n = 15)	P-value
Calm	2.83 → 3.33 (+0.50)	2.87 → 3.27 (+0.40)	2.42 → 2.92 (+0.50)	2.73 → 3.33 (+0.60)	0.967
Tense	2.33 → 1.50 (-0.83)	2.13 → 1.60 (-0.53)	2.58 → 1.33 (-1.25)	2.60 → 1.47 (-1.13)	0.498
Upset	1.83 → 1.17 (-0.67)	1.93 → 1.53 (-0.40)	2.33 → 1.50 (-0.83)	2.00 → 1.20 (-0.80)	0.828
Content	2.67 → 3.33 (+0.67)	2.47 → 2.80 (+0.33)	2.75 → 2.83 (+0.08)	2.53 → 2.93 (+0.40)	0.898
Worried	2.33 → 1.50 (-0.83)	2.00 → 1.47 (-0.53)	3.08 → 1.58 (-1.50)	2.53 → 1.40 (-1.13)	0.225

## Discussion

Multiple studies have confirmed the beneficial effect of classical music on anxiety in the hospital setting. Performing arts practices have also been used to support inclusion among disparate groups of people globally. The role of music-making has been shown to be beneficial in peacebuilding and healing among ex-combatants in Colombia. Social music projects, such as choirs for homeless people in Brazil, hip-hop contests with at-risk youth in Mexico, and collaborative songwriting with refugees in the UK, have all been demonstrated to increase the sense of well-being [19-21]. However, it is not a given that classical music is therapeutic in all settings. In fact, sound in general can be used to generate discomfort or create an environment of fear and dread. Sonic weapons have been used in psychological warfare against Manuel Noriega by the US Army in Panama in 1989 and against the Branch Davidians in Waco, Texas, by the FBI in 1993. During the conflict in 2009 in Gaza, the Israeli Air Force used low-altitude supersonic flights over the densely populated Gaza Strip, creating deafening sounds, shattering windows, shaking entire apartment buildings, and terrorizing civilians. These sonic booms had a widespread impact on residents, especially children, leading to severe psychological trauma and anxiety [22].

In less sinister forms, sound has been used in civilian control, such as blasting high-frequency deterrents against teenagers in public spaces. Sonic gentrification can mean banning loud street music and noise, as well as using sound to gentrify a neighborhood. In cities across America, retailers and public transit authorities are known to have used classical music [23], often music by Bach or Mozart played loudly, to discourage loitering in public or commercial spaces in various cities [24]. Sonic gentrification is in line with openly punitive urban policies and restrictive zoning laws that perpetuate exclusion [25]. We are in the midst of a housing crisis because of the intersection of affordability, income inequality, and scarcity. Instead of universal housing, the current focus has been on the management of homelessness through displacement or criminalization [26,27]. Playing classical music in these settings aims to create an uncomfortable or annoying environment to encourage unwanted groups to leave. The rationale for this tactic may stem from the fact that classical music is perceived as an “elite” genre of music. The hidden message of playing classical music is to mark a location as not for the homeless, thus increasing their discomfort and making them want to leave [28]. The tactic presumes that classical music is unappealing to this population and would have a different impact on their anxiety than on that of other populations, such as in the hospital setting, where classical music is frequently played because of its known beneficial effect on anxiety in this stressed population.

However, this study found that live classical music was associated with a reduction in self-reported anxiety in both the Hospital and Unhoused Groups. Review of the scientific literature to date suggests that this is a novel study demonstrating a beneficial effect of classical music on anxiety in the unhoused population. While both groups were found to have benefited from classical music, it is interesting to note differences between the Unhoused and Hospital Groups. There was a greater decrease in “worried” feelings in the Unhoused Group compared with the Hospital Group. This may have been because the Unhoused Group had higher baseline values for feeling “worried” and “upset.” It is perhaps not surprising, given the dramatic impact a lack of housing can be expected to have on one’s mood, that the unhoused participants had higher “worried” and “upset” feelings at baseline, allowing for greater potential to improve those feelings compared with the Hospital Group, in which participants did not report as high a level of those same feelings at baseline. The Unhoused Group was not asked whether they had previously experienced hearing classical music played in locations where the intent was to get them to leave. However, it is interesting that unhoused participants who reported that they were not familiar with classical music were more likely to report improved “upset” scores. Perhaps some of the Unhoused Group had recalled hearing classical music in places where it had been “weaponized,” and thus, hearing classical music affected these participants less favorably.

There were several limitations to our study. The unhoused population represents a stressed group of people, and this study suggests that performing live classical music for the unhoused can have beneficial effects on their anxiety. There was no control group in which participants were simply exposed to silence. The Hospital Group was also a heterogeneous group comprising staff, family members, and patients. However, this study also raises important questions that may guide future research. While this study showed that classical music had a beneficial effect for the unhoused, it is unclear why. Hearing the sound of classical music may have resulted in the observed effect, or perhaps the benefit was derived from having a free, live performance, and therefore, the audience felt an appreciation for the musician that translated into positive effects on their anxiety. In addition, playing classical music that is not repetitive and at a loud volume could potentially demonstrate a reduction in anxiety levels in the unhoused population as well. Additional confounding factors included the possibility that some of the benefits were partially derived from being in a social or meal setting.

Future directions include qualitative studies of homeless persons to determine how live classical music made them feel, as well as testing the effect on anxiety of playing recorded classical music. In addition, different genres of music should be played in both recorded and live formats to determine whether self-selected music could lead to additional improvement in anxiety levels. Furthermore, music-making instead of music listening could be tested to determine whether the former can produce a greater change in the anxiety level of homeless individuals. The conditions for future studies can be better controlled than in this observational study. Participants could be divided into multiple groups with defined periods of exposure to different genres of music.

## Conclusions

This observational study found that exposure to live classical music had a beneficial effect on short-term self-reported anxiety levels among unhoused individuals. A similar effect of live classical music was also observed in the hospital setting. In the future, studies should compare live versus recorded music, as well as determine whether a similar beneficial effect can be achieved with other genres of music.
